# Primary Dural Lymphoma Mimicking En Plaque Cerebellopontine Angle Meningioma

**DOI:** 10.1155/2021/2845995

**Published:** 2021-07-23

**Authors:** Mei Xu, Ashish Bains, Yuan Rong

**Affiliations:** ^1^Department of Biomedical Sciences, Philadelphia College of Osteopathic Medicine, Philadelphia, Pennsylvania, USA; ^2^Department of Pathology and Laboratory Medicine, Temple University Lewis Katz School of Medicine, Philadelphia, Pennsylvania, USA

## Abstract

Primary dural lymphoma (PDL) usually arises from the calvarial dura without the brain parenchyma or systemic involvement and thus may not be considered as a typical form of primary CNS lymphoma (PCNSL). It is exceedingly rare. When it occurs, it might not be suspected as a primary diagnosis on clinical and radiologic findings. We present a PDL case that occurs at the cerebellopontine (CP) angle mimicking en plaque meningioma. The tumor histopathology showed a lymphoproliferative disorder immunophenotypically consistent with a low-grade marginal zone lymphoma. Bone marrow and systemic involvements were not identified, and a diagnosis of PDL was established. As a residual tumor at the CP angle was inaccessible to surgery, postoperative radiation therapy was performed. No recurrence was found at 15-month follow-up. PDLs are mostly indolent and have a good prognosis. There is no doubt that the most important differential diagnosis is meningioma. Furthermore, the present case emphasizes the necessity of an intraoperative consultation and knowledge of this rare yet essential form of PCNSL so that appropriate studies can be ordered.

## 1. Introduction

PCNSL is an extranodal non-Hodgkin lymphoma that commonly involves the brain, spinal cord, leptomeninges, and ocular structures [1]. Dura involvement is typically secondary to the lesion in the brain parenchyma [2]. However, PDL is extra-axial and arises from the dura in the absence of systemic or brain parenchymal involvement [3]. Technically, it may not be best classified as PCNSL. PDL is in fact extremely rare and only comprises less than 1% of PCNSL [4]. A recent retrospective study of the database from Massachusetts General Hospital and Yale School of Medicine shows that 20 out of 316 PCNSL patients are PDL [5]. PDL usually presents as a low-grade marginal zone B-cell lymphoma, but other subtypes exist as well [5]. An extranodal marginal zone lymphoma usually occurs in a variety of extranodal sites. When it involves the lung, gastrointestinal tract, bladder, and lacrimal gland, it is known as a mucosa-associated lymphoid tissue lymphoma [6, 7]. Due to the fact that the majority of PDLs are low-grade marginal zone B-cell lymphomas, the prognosis tends to be much more favorable, especially in comparison to bona fide PCNSLs. Its clinical and radiographic features mostly mimic meningioma so that diagnosis of meningioma is often made clinically prior to the pathological findings [8]. Given the accumulating reports for the past decades, PDL histopathology is much more defined. Further exploration of variability of PDL would shed light on its pathogenic mechanism.

## 2. Case Presentation

**Figure 1:**
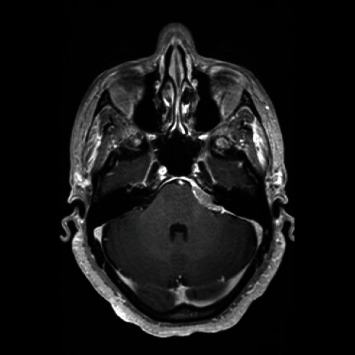
Post contrast MRI demonstrates a well-defined homogeneously enhancing extra-axial dural-based mass in the left CP angle suspicious for an en plaque meningioma.

**Figure 2:**
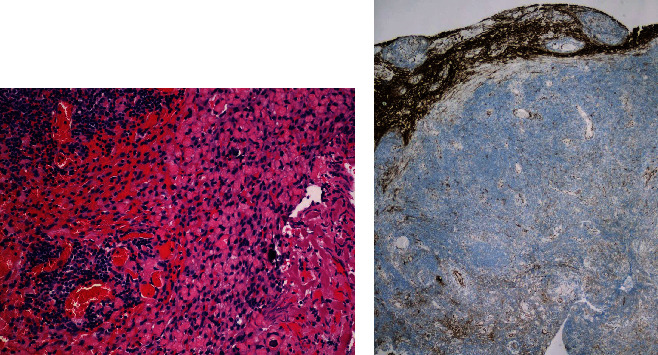
(a) Routine H&E stain demonstrates peripheral scattered meningothelial cells. (b) These meningothelial cells are highlighted by somatostatin receptor 2a immunohistochemistry.

**Figure 3:**
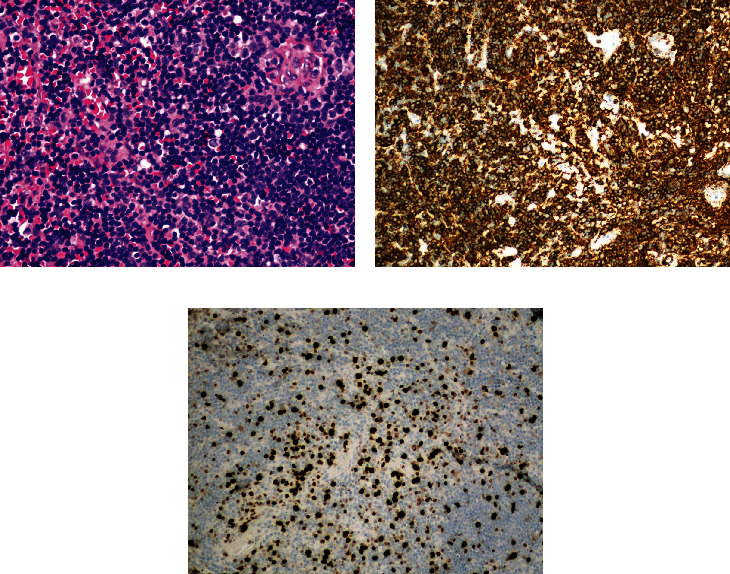
(a) Routine H&E stain demonstrates the atypical lymphocytes. These lymphocytes are positive for CD20 (b) with increased Ki-67 proliferation index (c).

A 67-year-old female patient was initially seen for left-sided facial and mouth numbness, watering eye, and altered taste, which progressed over one year. MRI of the brain with and without contrast revealed a 2.6 × 2.1 cm well-defined homogeneously enhancing extra-axial dural-based mass at the left CP angle, which extended superiorly to the tentorium, suspicious for en plaque meningioma (Figure 1). The patient was admitted to the neurosurgery department and received surgical resection of the tumor. External beam radiation with a total of 36 Gy over 20 fractions was used for the residual tumor, which was inaccessible at the CP angle during surgery. The histopathological analysis revealed a dense atypical small to intermediate lymphocytic infiltrate with a focal nodular pattern. Focal meningothelial cells demonstrated by epithelial membrane antigen, progesterone receptor, and somatostatin receptor 2a immunohistochemistry were primarily present at the periphery (Figure 2). Immunohistochemistry for CD68 and CD138 highlighted scattered histiocytes and plasma cells and CD21 and CD23 for follicular dendritic cells. The atypical lymphocytes were positive for CD20, PAX-5, and Bcl-2, but negative for CD5, CD10, Bcl-6, CD1a, ALK, and cyclin D1. The Ki-67 proliferation index was 20-30% (Figure 3). In situ hybridization to Epstein-Barr-encoded RNA was negative. Clonal rearrangements of the immunoglobulin heavy chain and kappa light chain genes were detected by PCR. These results were consistent with B-cell lymphoproliferative disorder. Bone marrow aspiration biopsy and peripheral flow cytometry were negative. Clinically, no systemic lymphoma was identified. Cytogenetics demonstrated a normal karyotype. The patient was diagnosed with a primary dural low-grade extranodal marginal zone B-cell lymphoma. She was in remission without recurrence at the 15-month follow-up.

## 3. Discussion

PDLs grow from the dura without the brain parenchymal or systemic involvement. The lymphomas infiltrate meningeal tissue, which often presents with sclerotic features. Most of PDLs arise from the calvarial dura. According to Karschnia et al., 55% are from the calvarial dura, 30% from the skull base, and 15% from the spinal dura out of 20 PDL cases in the database [5]. They rarely occur at the CP angle. This case showed an extra-axial dural-based mass at the left CP angle. The tumor extended superiorly to the tentorium and resulted in trigeminal nerve palsy. It was not completely accessible to surgery, so that postoperative radiation therapy was performed in the surgical bed. The majority of PDLs are low-grade marginal zone B-cell lymphomas [5, 9]. They respond well to radiation and tend to have indolent clinical courses [3, 10]. This is quite consistent with marginal zone B-cell lymphomas in other sites, such as the stomach and thyroid [10]. Although local and systemic relapses can occur, they still respond to subsequent treatment [9]. Immunohistochemical analysis of PDLs often reveals features of marginal zone B-cells, e.g., CD20+, CD5-, CD10-, and Bcl-2+, that help to distinguish them from follicular B-cells. PDLs frequently present similar clinical features and neuroimaging findings to meningioma, such as age of onset and higher occurrence in women and isointense extra-axial masses on radiographs. Therefore, histopathological analysis is critical for PDL diagnosis.

PDL is rare and usually has a different prognosis from PCNSL. Surgical excision has been established as the standard treatment for the past decades. However, complete resection could be a challenge if PDL presents as an infiltrative and/or en plaque lesion. Adjuvant treatment with either radiation or chemotherapy is then indicated. In this case, postoperative external beam radiation to the residual tumor allowed complete remission. Since recurrence can develop after complete remission following the initial treatment, regular follow-up is highly recommended [9]. Although most of cerebral PDLs are low-grade marginal zone B-cell lymphomas, other subtypes, including diffuse large B-cell, follicular, T-cell non-Hodgkin, and B-cell small lymphocytic lymphomas, have been reported [5, 11]. Spinal PDLs, however, are frequently diffuse large B-cell lymphomas [12]. So far, no definitive adjuvant treatment is established for all subtypes. Unquestionably, a large database is needed to explore the significant association between the treatment options and their clinical outcomes that appear to be well based on the published data so far and likewise in this case. Besides, investigation of pathogenic development of PDLs might lead to novel therapeutic approaches.
